# Multiscale fluorescent tracking of immune cells in the liver with a highly biocompatible far-red emitting polymer probe

**DOI:** 10.1038/s41598-020-74621-9

**Published:** 2020-10-16

**Authors:** Malo Daniel, Laurence Dubreil, Romain Fleurisson, Jean-Paul Judor, Timothée Bresson, Sophie Brouard, Arnaud Favier, Marie-Thérèse Charreyre, Sophie Conchon

**Affiliations:** 1 Université de Nantes, INSERM, UMR1064, Centre de Recherche en Transplantation et Immunologie, ITUN, 44000 Nantes, France; 2 PAnTher, INRAE, École nationale vétérinaire, agro-alimentaire et de l’alimentation Nantes-Atlantique (Oniris), Université Bretagne Loire (UBL), 44307 Nantes, France; 3 Laboratoire Ingénierie des Polymères (IMP), CNRS UMR5223, Université Lyon1, Université de Lyon, Lyon, France

**Keywords:** Multiphoton microscopy, Sensors and probes, Imaging the immune system, Hepatology

## Abstract

The development of innovative immune cell therapies relies on efficient cell tracking strategies. For this, multiscale fluorescence-based analyses of transferred cells into the host with complementary techniques, including flow cytometry for high-throughput cell analysis and two-photon microscopy for deep tissue imaging would be highly beneficial. Ideally, cells should be labelled with a single fluorescent probe combining all the properties required for these different techniques. Due to the intrinsic autofluorescence of most tissues and especially the liver, far-red emission is also an important asset. However, the development of far-red emitting probes suitable for two-photon microscopy and compatible with clearing methods to track labelled immune cells in thick samples, remains challenging. A newly-designed water-soluble far-red emitting polymer probe, 19K-6H, with a large Stokes shift, was thus evaluated for the tracking of primary immune CD8 T cells. These cells, prepared from mouse spleen, were efficiently labelled with the 19K-6H probe, which was internalized via endocytosis and was highly biocompatible at concentrations up to 20 μM. Labelled primary CD8 T cells were detectable in culture by both confocal and two-photon microscopy as well as flow cytometry, even after 3 days of active proliferation. Finally, 19K-6H-labelled primary CD8 T cells were injected to mice in a classical model of immune mediated hepatitis. The efficient tracking of the transferred cells in the liver by flow cytometry (on purified non-parenchymal cells) and by two-photon microscopy on 800 μm thick cleared sections, demonstrated the versatility of the 19K-6H probe.

## Introduction

Immune cell-based therapy is today considered as a major avenue for the regulation of immune responses in pathological situations such as cancer^1^ or transplantation^2,3^. In this context, the development of in vivo tracking of primary immune cells in animal model is of paramount importance to better understand the migration and behaviour of implanted cells in the host^4^. The main challenge in this field is to efficiently discriminate transferred cells from host tissue thanks to multiscale techniques including microscopy and flow cytometry. Therefore, optical strategies based on fluorescence detection have been developed, ranging from genetic engineering to exogenous labelling^5^. For this later purpose, various fluorescent probes have been designed for the labelling of immune cells prior to their engraftment^6^. Their most important properties should be: biocompatibility, as primary cells are fragile and difficult to maintain in an unaltered state prior to transfer; brightness and stability of the fluorescent signal, to ensure an efficient visualisation of the labelled cells over time. The range of absorption and emission wavelengths of the fluorescent probe is also critical considering the possible autofluorescence of the imaged tissue and the type of microscopy implemented for the tracking^7^. Fluorescence microscopy observations are often complicated by tissue autofluorescence, which mainly occurs in the UV and visible ranges, making far-red (650–750 nm) and near-infrared (750–950 nm) emission wavelengths highly attractive^8–10^. Moreover, when deep-tissue imaging of the transferred cells is desired, it is relevant to switch from one- to two-photon (2P) excited fluorescence (TPEF) microscopy^11,12^.

TPEF-microscopy enables deep tissue imaging, as the near-infrared excitation by a pulsed laser induces less absorption and scattering in biological samples compared to the continuous visible light used in widefield and confocal microscopy^13^. The 2P-excitation wavelength being almost twice the one-photon excitation value, it is very easy to efficiently separate the emitted fluorescence signal from the excitation light with appropriate filters. Another advantage of TPEF-microscopy is the low out-of-focus phototoxicity thanks to a highly localized excitation volume (~ 0.1 μm^3^) at the focal point of the laser and thus the high compatibility with in vivo biological analysis^14,15^. In addition, no parasite fluorescence signal is generated below and above the focal plane. In order to further increase imaging penetration depth, several clearing protocols have been developed^16^. Most of them are based on tissue refractive index homogenization, and the resulting high tissue transparency drastically reduces light scattering in biological samples. Clearing protocol coupled with TPEF-microscopy enables an imaging depth of up to 4 mm in most tissues which is highly attractive to study tissue complexity and to image rare events.

One remaining challenge is to reconcile far-red fluorescence emission with a high 2P-absorption for bioimaging. Indeed, most of the common far-red emitting fluorophores are efficiently excited in the 600–700 nm wavelength range, thus implying an optimal 2P-absorption above 1200 nm which is currently out of the range of standard femto-second pulse laser used on TPEF-microscopes used in core facilities. Therefore, fluorescent probes exhibiting a large Stokes shift (with excitation around 500 nm and emission in the far-red range) as well as high 2P-absorption cross section values are highly desirable.

Far-red emitting nanoparticules^17^ have been synthesized in the last decade but with limited evidence of biocompatibility in primary cells or TPEF-microscopy applications. For instance, cell labelling with quantum dots (QDs), fluorescent inorganic semiconductor nanoparticules displaying unique optical features depending on their size, has recently emerged^18^. However, their cytotoxicity, mainly due to the liberation of free ions, is now well documented^19,20^. A new generation of far-red emitting QDs has been recently described to efficiently label breast cancer cell line in culture without cytotoxicity^21^. Primary cell or in vivo imaging and TPEF-microscopy applications have not been reported yet. Organic semiconducting polymer nanoparticules or dots (Pdots) represent the main competitor of QDs with an expected low intrinsic cytotoxicity^22^. Far-red emitting Pdots have been used for in vivo tumor imaging in mouse brain by confocal microscopy^23^ and very recently for multiphoton imaging of microvasculature in mice^24^. However, Pdots have not been used so far to label primary cells and their biocompatibility remains to be thoroughly investigated. Harmonic nanoparticles have been already described as powerful tool for muscle stem cell tracking in tissue^25^ but currently there is no flow cytometer capable to detect second harmonic generation. It is the reason why we have focused our interest on a different family of polymer probes offering a versatile alternative, compatible with both flow cytometry and various types of fluorescence microscopy investigations.

Such polymer probes are not nanoparticles but water-soluble polymer chains^26^, bearing several far-red emitting fluorophores^27^ exhibiting large Stokes shifts (around 180 nm) and high 2P-cross section values (around 440 GM in water for a similar fluorophore surrounded by a star-shaped polymer shell)^28^ for an efficient 2P-absorption. They have a controlled chain structure (molecular weight, dispersity of the chains, number of fluorophores per chain, possible presence of an anchoring group like a lipid). Previous results demonstrated an efficient labelling of enveloped viruses^29^, living cells and zebrafish embryos^30^ with convenient TPEF-imaging and reported a low cytotoxicity on Jurkat and HeLa cell lines^30^. However, up until now, these polymer probes have not been used to label primary cells.

**Figure 1:**
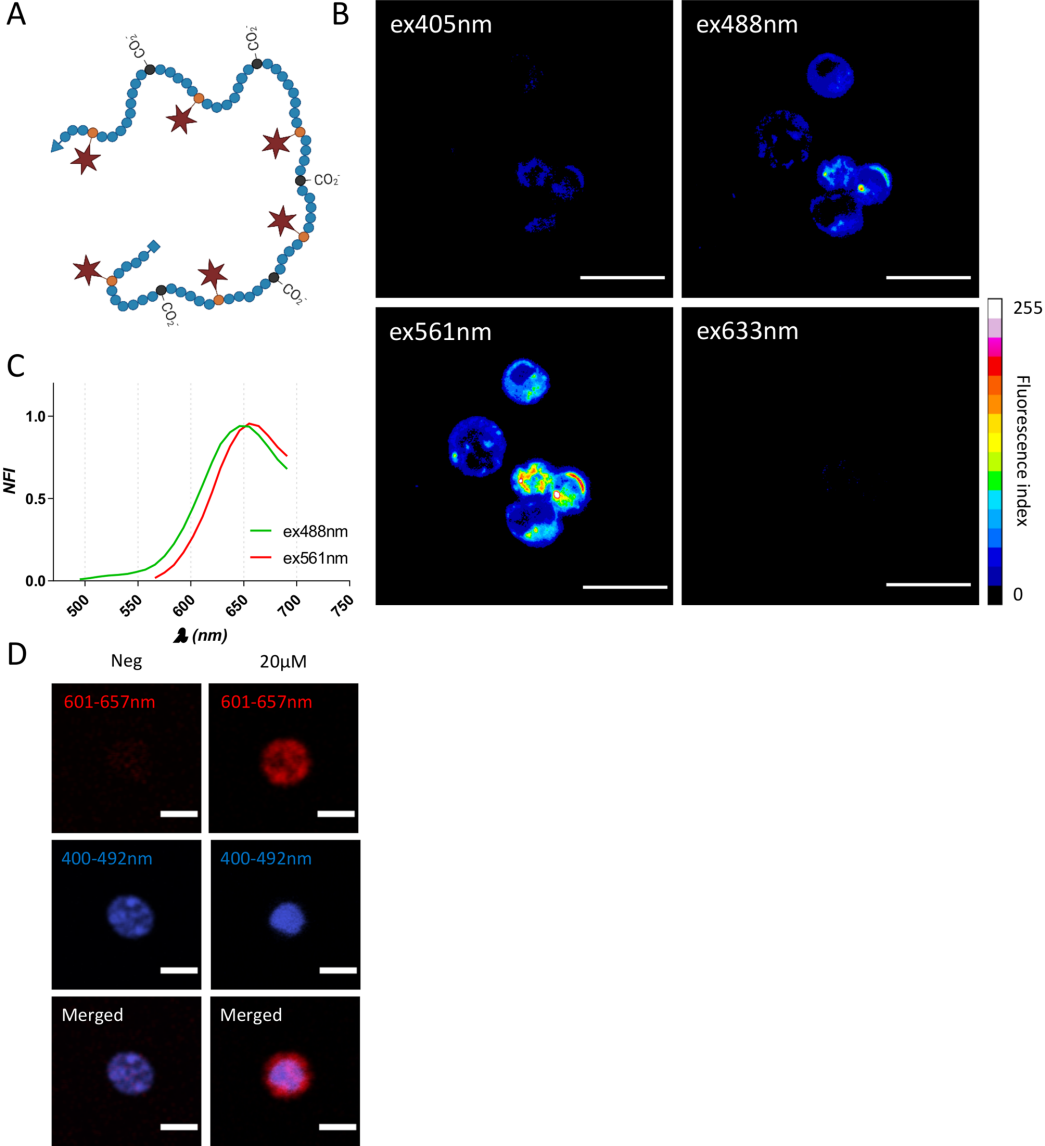
(**A**) Schematic representation of the 19K-6H polymer probe. (**B**) Confocal spectral imaging (objective ×63) of living pCD8 T cells labelled with 19K-6H probe (20 μM, 15-h incubation) from excitation at 405 nm, 488 nm, 561 nm and 633 nm. Each image is a representation of maximum intensity projection obtained between 566 and 700 nm. Grey levels (8bits) were color-coded with the 16 colors look-up-table (LUT) from FIJI. Scale bar: 10 μm. (**C**) Emission spectra of 19K-6H in cellulo, from 488 and 561 nm excitation, determined with spectral imaging (fluorescence intensity collected every 8.9 nm between 566 and 700 nm). (**D**) TPEF-imaging (objective ×25) of living pCD8 T cells counterstained with Hoechst nuclear probe, labelled with 19K-6H probe (20 μM, 15-h incubation) (top panel) compared to negative control (bottom panel, only Hoechst nuclear probe). Dual sequential excitation at 900 nm and 1040 nm for respective observation of nuclear Hoechst staining (blue channel, 400–492 nm) and internalized 19K-6H probe (red channel, 601–657 nm). Fluorescent images (visualized in blue and red, false colors) are presented separately on left and middle panel and merged on right panel. Scale bar: 5 μm.

In this article, we investigated the newly designed far-red emitting polymer probe 19K-6H for in vivo tracking of murine primary CD8 T lymphocytes (pCD8 T cells). This polymer probe bears an average of 6 fluorophores per chain and exhibits numerous carboxylate groups (up to 45 per chain) that contribute to its water-solubility. As opposed to previous probes of the same family, the chain is not terminated by a lipid group since preliminary tests showed that this structural characteristic was not necessary to get an efficient labelling of lymphocytes. The hydrodynamic diameter of this polymer probe that adopts a coil conformation in aqueous solution is estimated to be in the range of 5–8 nm^31^ (schematic representation in Fig. 1A, chemical structure in Figure S1). The absorption and fluorescence emission spectra of the polymer probe in water are provided in Figure S2.

We first thoroughly characterized the optical properties, the biocompatibility and the internalization route of the 19K-6H probe in primary CD8 T cells. Then, we carried out evaluation of the 19K-6H probe for tracking the labelled primary CD8 T cells in a mouse model of immune cell infiltration in the liver parenchyma, by confocal and TPEF-microscopy as well as by flow cytometry.

## Material and methods

### 19K-6H polymer probe synthesis

All chemicals were purchased from Sigma-Aldrich at the highest purity available and used without further purification. Solvents were used as received, either from Fisher Scientific or Acros Organic. Poly(*N*-acryloylmorpholine-*co*-*N*-acryloxysuccinimide), P(NAM-*co*-NAS), copolymer chains (number average molecular weight = 19,400 g mol^−1^, dispersity = 1.07) have been synthesized from a *tert*-butyl dithiobenzoate RAFT agent according to a previously reported process^32^. The organic fluorophore (isophorone derivative) has already been described^27^.

The synthesis of the 19K-6H polymer probe was performed as follows: 25.1 mg of P(NAM-*co*-NAS) copolymer were dissolved in 1 mL of chloroform in a round bottom flask equipped with a magnetic stirrer. Fluorophore (3.7 mg, dissolved in 0.5 mL of chloroform) was added together with 2 molar equivalents of triethylamine (0.98 mg). Polymer concentration was adjusted to 10 mg mL^−1^ with chloroform. The reaction was carried out at 30 °C in the dark under stirring for 2 h. The binding yield was followed by size exclusion chromatography (SEC) with a UV–Visible detector according to a previously described method^33^ and reached 75% (corresponding to an average of 6 fluorophores per polymer chain). In order to eliminate the residual fluorophores, the mixture was precipitated in a large volume of diethyl ether and the fluorescent polymer was recovered as a powder by centrifugation (10,000 t min^−1^, 10 min, 4 °C). The procedure was repeated until full discoloration of the supernatant. The fluorescent polymer was finally dried under vacuum.

Hydrolysis of the residual succinimide ester units along the polymer chain was carried out using 50 mL of borate buffer (50 mM, pH = 9.3) directly added to 25 mg of the fluorescent polymer, and let at room temperature under stirring for 48 h. Then, the fluorescent polymer was purified by dialysis (SPECTRUM LABS, Spectra/Por 6, MWCO: 2000 g mol^−1^) against deionized water (2 baths) and milliQ water (1 bath). The dark-red-colored fluorescent polymer was dried by lyophilization. After this hydrolysis step, the polymer probe exhibits numerous carboxylate groups (–COO^**–**^) along the polymer chain: a maximum of 45 per chain (depending on the pH and on the distribution of these groups). Consequently, the molecular weight of the polymer chain (under the –COONa form) decreased to 17,500 g mol^−1^.

### Characterization of the 19K-6H polymer probe

Size exclusion chromatography (with a UV–Visible detector), absorption and fluorescence emission spectra were recorded as previously described^26^. The molar extinction coefficient (ε) of the polymer probe and its fluorescence quantum yield (ϕ) in water were determined, following a previously described protocol^33^, from the absorption and emission spectra, respectively (ε  = 43,400 ± 700 mol^−1^ L cm^−1^ at λ_abs max_ = 504 nm; ϕ  = 0.064 at λ_ex_ = 510 nm). Reference was erythrosin B in methanol (ϕ  = 0.09 at λ_ex_ = 510 nm). Therefore, the brightness (ε·ϕ) of the polymer probe in water was calculated to be 2800 mol^−1^ L cm^−1^.

### Mice

Male C57Bl/6 mice were purchased from JANVIER LABS (France), used between 7 and 12 weeks of age and housed at the UTE IRS-UN *(Nantes–France)* animal facilities. Mice were fed ad libitum and allowed continuous access to tap water. All procedures were approved by the regional ethical committee for animal care and use and by the French Ministry of Research (agreement APAFIS #13742). All experiments were performed in accordance with relevant guidelines and regulations.

### Tissue and cell preparations

Livers were PFA-fixed for 48 h or included in OCT Compound (TISSUE-TEK) and frozen in liquid nitrogen (− 196 °C)-cooled isopentane after in vivo elimination of blood by perfusion of HBSS 1× buffer (GIBCO). For confocal imaging, frozen liver samples were acetone-fixed and cryo-sectioned at 15 μm then analysed on the laser confocal scanning microscope LSM780 ZEISS (CARL ZEISS MICROSCOPY, Jena, Germany). For multiphoton imaging, PFA-fixed liver samples were sectioned with a scalpel to get 0.8–1 mm thick sections then cleared by using CUBIC protocol and analysed on the A1R-MP NIKON multiphoton microscope *(technical parameters below).*

Liver non-parenchymal cells (NPC) were isolated as previously described^34^. Briefly, after perfusion with HBSS 1× buffer (GIBCO), livers were digested with collagenase IV (SIGMA-ALDRICH—C5138) and NPC enriched by Percoll (GE HEALTHCARE—17-0891-01) density gradient centrifugation and red blood cells lysis.

pCD8 T cells used in biocompatibility, labelling study and adoptive transfer experiments were isolated from C57Bl/6 mice spleen after red blood lysis (ACK buffer: NH_4_Cl 155 mM, KHCO_3_ 10 mM EDTA 0.1 mM) and negatively sorted with MILTENYI CD8a^+^ T cell isolation kit (130-104-075).

### CUBIC clearing

Clearing of 800 μm PFA-fixed sections were performed with the CUBIC Protocol I for Adult Mouse Organ Samples and Marmoset Brain described by Tainaka et al.^35^. PFA-fixed livers were washed with gentle shaking in PBS for 15 h at room temperature then incubated in CUBIC-L (TCI − T3740) for 4 days with gentle shaking at 37 °C. After another PBS wash, organ sections were incubated with 1:1 water-diluted CUBIC-R+ (TCI – T3741) with gentle shaking for 1 day at room temperature then in CUBIC-R+ for one more day in the same conditions.

### In vitro biocompatibility assay

pCD8 cells were cultured during 5 days (from Day 0 to Day 4) in RPMI medium (GIBCO—31870074) supplemented with 10% FBS, 1% Penicillin/Streptomycin, 1% Glutamine, 10 mM Hepes (GIBCO—15630056), 1 mM Sodium Pyruvate (GIBCO—11360039), 1× Non-Essential Aminoacids (GIBCO—11140035) and 50 μM 2-mercaptoethanol (SIGMA-ALDRICH—M6250). From Day 0 to Day 1, pCD8 T cells were incubated with increasing concentrations of 19K-6H probe (0; 0.5; 5; 20 μM). On Day 1, cells were washed and resuspended in complete RPMI medium, with IL-2 (CELLGENIX 10 U mL^−1^) and T activator CD3/CD28 Dynabeads (GIBCO—ratio cell/bead 1:1) to induce their proliferation. Cells were analysed on LSR II flow cytometer (BECTON DICKINSON). Viability and proliferation were monitored with the LIVE/DEAD Fixable Yellow Dead Cell Stain Kit (INVITROGEN) and 123 count eBeads Counting Beads (INVITROGEN), respectively. The cell activation state was analysed by flow cytometry with antibodies directed towards early activation marker CD69 (FITC—BD 553236), and late activation markers CD44 (APC—BD 559250) and CD25 (PE—BD 553866).

### Characterization of 19K-6H labelling on primary immune cells

pCD8 T cells were prepared as described above. From Day 1 to Day 4, cells were plated in chambered coverslips (IBIDI μ-Slide 18 well—81826—15,000 cells/well) coated with Poly-l-Lysine (SIGMA-ALDRICH—P4707) then imaged on the laser confocal scanning microscope LSM780 ZEISS (CARL ZEISS MICROSCOPY, Jena, Germany) at 63× magnification. Transmission light was used to localize the cells and laser line 561 nm to analyse the 19K-6H fluorescence. Spectral detection was performed with ZEISS 32 Channel GaAsP spectral detectors and images analysed with FIJI image analyse software. A minimum of 100 cells were imaged for each condition.

### Internalization pathway investigation

HeLa cells (ATCC CCL-2) were cultured in DMEM medium (GIBCO—11960085) supplemented with 10% FBS, 1% Penicillin/Streptomycin and 1% Glutamine. The cells (50% of confluency) were transfected with 100 ng of Rab5a-mCherry^36^ or Rab7-GFP^37^ plasmids, preincubated with 0.5 μL Lipofectamine (INVITROGEN—15338100), during 24 h at 37 °C in complete DMEM as described above (without Penicillin/streptomycin). Cells were then washed and plated in chambered coverslips (IBIDI μ-Slide 18 well—81826—3000 cells/well) and allowed to adhere overnight. Nuclei were counterstained with Hoechst 33342 (THERMOFISHER—H1399) diluted at 1:2000 in complete DMEM medium during 10 min at 37 °C. Medium was carefully replaced with complete DMEM containing 10 μM of 19K-6H probe right before imaging. Confocal imaging was performed with the laser confocal scanning microscope LSM780 ZEISS (CARL ZEISS MICROSCOPY, Jena, Germany) at 63× magnification. 405 nm, 488 nm and 561 nm laser lines was used to excite Hoechst 33342, GFP, mCherry and 19K-6H probe, respectively. Spectral detection was performed with ZEISS 32 Channel GaAsP spectral detectors and images analysed with FIJI image analyse software.

Inhibition of endocytosis was performed by pre-incubating HeLa cells or pCD8 T cells at 4 °C or in presence of 80 μM Dynasore^38^ (SIGMA-ALDRICH—D7693) or DMSO (Dynasore-vehicle—SIGMA-ALDRICH—276855) for 1 h. Cells were then incubated 8 h with 19K-6H probe, at 4 °C or in presence of 80 μM Dynasore or DMSO. 19K-6H internalization was assessed by flow cytometry.

### pCD8 T cell adoptive transfer

pCD8 T cell isolated from C57Bl/6 mice were incubated overnight in complete RPMI medium, with IL-2 (CELLGENIX 10 U mL^−1^) and activator antibodies : 1 μg mL^−1^ anti-CD3 (BD 553057—*coated*) and 1 μg mL^−1^ anti-CD28 (BD 553294), with or without 20 μM of 19K-6H probe (1.5 × 10^7^ cells and 0.5 × 10^7^ cells respectively). The next day, cells are washed in PBS 1× then injected *i.v.* in 2 C57Bl/6 mice for systemic delivery. Recipient mice are then injected *i.v.* with 15 mg kg^−1^ Concanavalin A (SIGMA-ALDRICH—C2010) to induce a T cell-mediated acute liver hepatitis^39^. Mice were then sacrificed and livers and NPC were prepared as described above. Tissues were analysed by both confocal and TPEF-microscopy as described above. Cells were analysed on LSR II flow cytometer (BECTON DICKINSON) with antibodies directed towards CD3 (V450—BD 560801), CD8 (APC—BD 553035) and CD69 (FITC—BD 553236) markers. 19K-6H fluorescence was detected in the Pe-Cy5.5 channel (λ_ex_ 561 nm, λ_em_ 710/50 nm).

### Confocal microscopy

The inverted laser scanning microscope LSM780 ZEISS (CARL ZEISS MICROSCOPY, Jena, Germany) was equipped with solid state lasers 405, 561 and 633 nm and argon laser 455, 488, 514 nm and ZEISS 32 Channel GaAsP spectral detectors. Spectral sequences of 32 images were obtained using 8 nm band pass filters in the 405–700 nm range. Linear unmixing process of data obtained from spectral imaging was performed for matching the spectral variations in the lambda stack of the cells labelled with the 19K-6H probe and autofluorescence spectra recorded from control specimen (unstained cells and non-injected liver). The objectives used were Immersion 63X objective lens (NA 1.4 Oil DIC Plan-Apochromat) and 20× objective lens (NA 0.8 Plan-Apochromat).

### Two-photon imaging

The A1R-MP NIKON microscope was equipped with an Insight Deepsee laser from SPECTRA-PHYSICS, tunable in the 680–1300 nm range, < 120 fs pulse width with a dual output at 1040 nm for simultaneous two-photon imaging. The system was equipped with three high sensitive channels GaAsp Non Descanned Detectors (NDD) and one supplementary channel PMT NDD. Auto laser alignment was performed when changing multiphoton excitation wavelength. The configuration of the filters attached to NDD were (1) band-width 400–492 nm, (2) band-width (500–550 nm), (3) band-width (563–588 nm), (4) band-width (601–657 nm). The immersion objective used was an apochromat 25× MP1300 objective lens (NA 1.10, WD 2.0 mm).

## Results

### Fluorescence imaging of murine primary CD8 T cells labelled with 19K-6H probe

The synthesis and characterization of the 19K-6H polymer probe (Fig. 1A) are presented in the “Material and methods” section. The labelling of murine primary CD8^+^ T lymphocytes (pCD8 T cells) isolated from C57Bl/6 mice spleen with the 19K-6H probe was first assessed by fluorescence imaging. Freshly isolated pCD8 T cells were incubated overnight (15 h) with 20 μM of the 19K-6H polymer probe and imaged by confocal spectral microscopy. Four different excitation wavelengths (405, 488, 561, 633 nm) were tested to identify the best excitation/emission wavelengths for the visualisation of the probe (Fig. 1B). Emission spectra were recorded for the 488 nm and 561 nm excitation wavelengths and showed emission peaks at 650 nm and 660 nm, respectively (Fig. 1C). Spectral acquisitions for the 561 nm excitation wavelength that provided the highest fluorescence signal (Fig. 1B) confirmed a maximal emission around 650 nm (Figure S3), and this excitation was chosen for further confocal microscopy analyses. Morphologically, the 19K-6H signal appears intracytoplasmic and displays a specific sub-cellular localisation profile, with spots of high fluorescence intensity (diameter: 0.86 μm ± 0.55), suggesting an accumulation in vesicles. TPEF-microscopy on labelled primary CD8 T cells was performed after an overnight incubation with 20 μM of 19K-6H probe then nuclear counterstained. The 1040 nm excitation wavelength gave the best signal-to-noise ratio (data not shown) and resulted in strong fluorescence signal in the red/NIR channel around the nucleus of the 19K-6H labelled cells (Fig. 1C) with an absence of signal in the negative control (pCD8 T cells without 19K-6H probe, Fig. 1D).

### Stability of 19K-6H labelling in primary immune cells

**Figure 2:**
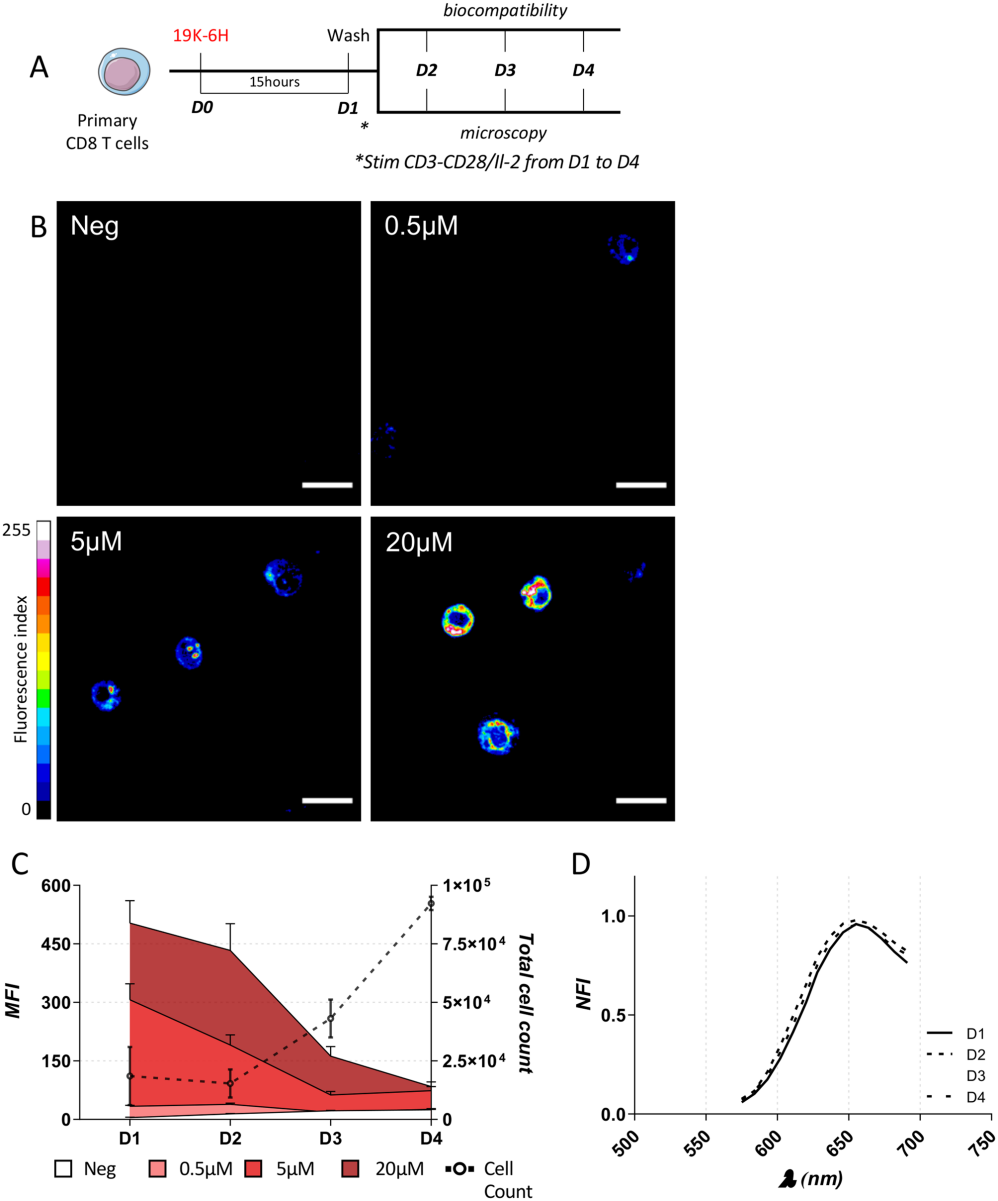
(**A**) pCD8 T cells are incubated overnight with 19K-6H probe then washed and resuspended in complete medium with stimulating signal. 19K-6H labelling and biocompatibility are analysed by confocal microscopy and flow cytometry respectively, from day 1 (D1) to day 4 (D4) post-incubation. (**B**) Fluorescence confocal microscopy images (objective ×63) of pCD8 T cells (λ_ex_ 561 nm), after 15 h (D1) incubation with increasing concentrations of 19K-6H probe (0 μM, 0.5 μM, 5 μM, 20 μM). Grey levels (8bits) were color-coded with the 16 colors look-up-table (LUT) from FIJI. Scale bar: 10 μm. (**C**) Evolution of 19K-6H labelling on proliferating pCD8 T cells from day 1 (D1) to day 4 (D4) after 15 h incubation of the 19K-6H probe at different concentrations. Mean Fluorescence intensity was measured on 100 to 1000 cells by condition. (**D**) Emission spectra of 19K-6H in cellulo on proliferating pCD8 T cells, D1, D2, D3 and D4 after cells labelling. MFI: mean fluorescence intensity; NFI: normalized fluorescence intensity.

The stability of the primary cell labelling with the 19K-6H probe was assessed in conditions of cell proliferation. pCD8 T cells isolated from splenocytes of C57Bl/6 mice were incubated for 15 h with increasing concentrations of 19K-6H probe (0 to 20 μM). Cells were then washed and cultured for 4 days (from Day 1 to Day 4) in the presence of stimulatory signals (anti-CD3/anti-CD28 agonists and Interleukin-2 (IL-2)) to induce their activation and proliferation (Fig. 2A). The 19K-6H labelling was assessed by confocal spectral microscopy at 561 nm excitation from Day 1 (before activation) to Day 4, then analysed with the FIJI software. First, after a 15-h incubation at the highest probe concentrations (20 and 5 μM), 95% of cells were labelled, whereas only 30% were labelled for the 0.5 μM concentration *(data not shown)*. The intracellular fluorescence increased proportionally from 0.5 to 5 μM (MFI from 33.61 ± 2.81 to 306.5 ± 41.39), and reached a maximum for the 20 μM condition (MFI: 503 ± 57.89 (Fig. 2B). From Day 1 to Day 4, in the presence of anti-CD3/anti-CD28 agonists and IL-2, a progressive decrease of the 19K-6H-labelled pCD8 T cell fluorescence was observed, concurrently with their proliferation (Fig. 2C, Figure S4). On Day 4, while pCD8 T cells had divided at least twice, those labelled with 5 μM and 20 μM of 19K-6H probe were still detectable (MFI: 73.80 ± 10.21 and 84.4 ± 11.94 respectively). These results were confirmed by flow cytometry experiments (Figure S5). Spectral microscopy analysis of the fluorescence within cells demonstrated the very stable emission profile of the 19K-6H probe, with identical emission spectrum over time (Fig. 2D). These results showed that the 19K-6H probe, used at 5 μM to 20 μM concentrations, optimally labelled pCD8 T cells and enable detection even after 3 days of active proliferation. Moreover, no events indicative of a potential cytotoxicity (e.g. plasma membrane blebbing, cell shrinkage) were observed, even at the highest 19K-6H concentration tested (20 μM).

### 19K-6H polymer probe biocompatibility

**Figure 3:**
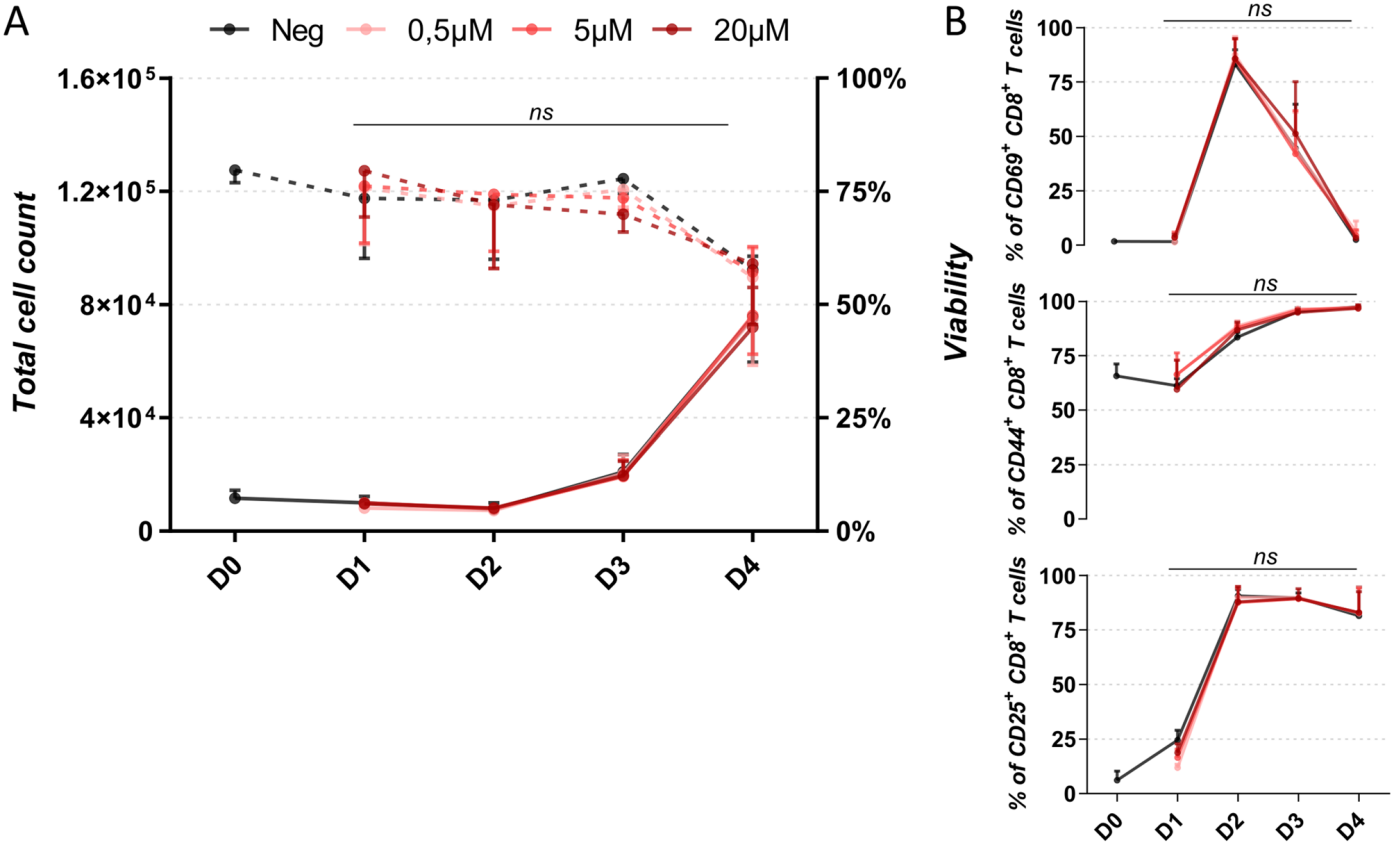
(**A**) Evolution of proliferation and viability of pCD8 T cells after 15 h incubation with increasing concentrations (0.5, 5, 20 μM) of 19K-6H probe compared to negative control (w/o 19K-6H). Full line: total cell count (left Y axis); dotted line: percentage of living cells (right Y axis). (**B**) Evolution of the expression of CD69, CD44 and CD25 activation markers. n = 3. The error bars indicate the standard deviation calculated for the averages from the three experiments. Significance is calculated by Two-Way Anova test; ns: non-significant.

Following the same experimental protocol (Fig. 2A), the innocuity of the 19K-6H polymer probe was further investigated on the cellular biology of pCD8 T cells isolated from the spleen of C57Bl/6. The proliferation, viability and expression of activation markers were assessed by flow cytometry from Day 0 (before adding 19K-6H) to Day 4. The 19K-6H polymer probe did not induce either short-term or long-term cell mortality. More than 70% of the cells remained alive 15 h post-incubation (Day 1) with 19K-6H probe, even at the highest 20 μM concentration, similarly to what was measured for the primary cells incubated without the probe (Fig. 3A).

In addition, the 19K-6H probe did not alter pCD8 T cells proliferation in response to the mitogenic signals. After 4 days of culture, the viability and proliferation of pCD8 T cells shared the same trend, independently of the 19K-6H concentration, with approximately 8 × 10^5^ cells and 60% of cell viability (no statistical differences).

Potential effect of 19K-6H probe on cell activation was studied before and after adding mitogenic signals (i.e. anti-CD3/anti-CD28 agonists and IL-2) in the culture medium. Expression of CD69, an early and transient activation marker, and of CD44 and CD25, late activation markers, were analysed at different time points by flow cytometry (Fig. 3B, and detailed histograms in Figure S6). After a 15-h incubation with the 19K-6H probe from 0 to 20 μM, there was no significant difference in the expression of activation markers by pCD8 T cells. In line with what was previously observed by confocal microscopy for cell viability and proliferation, the 19K-6H probe did not alter the activation of pCD8 T cells in response to mitogenic factors. For each tested concentration, the expression of CD69 marker in pCD8 T cells reached a peak of 80% by Day 2 and then progressively decreased to the basal level by Day 4. CD44 and CD25 were expressed respectively by 95% and 80% of cells by Day 4, comparable to what was measured for cells incubated without the probe.

Altogether, these results demonstrated that a 15-h incubation of pCD8 T cells with the 19K-6H probe did not induce short- or long-term cytotoxicity or cell activation, even at 20 μM. In addition, the 19K-6H probe did not disrupt nor alter the activation and proliferation abilities of the pCD8 T cells in response to mitogenic signals for 3 days.

### 19K-6H polymer probe internalization pathway

**Figure 4:**
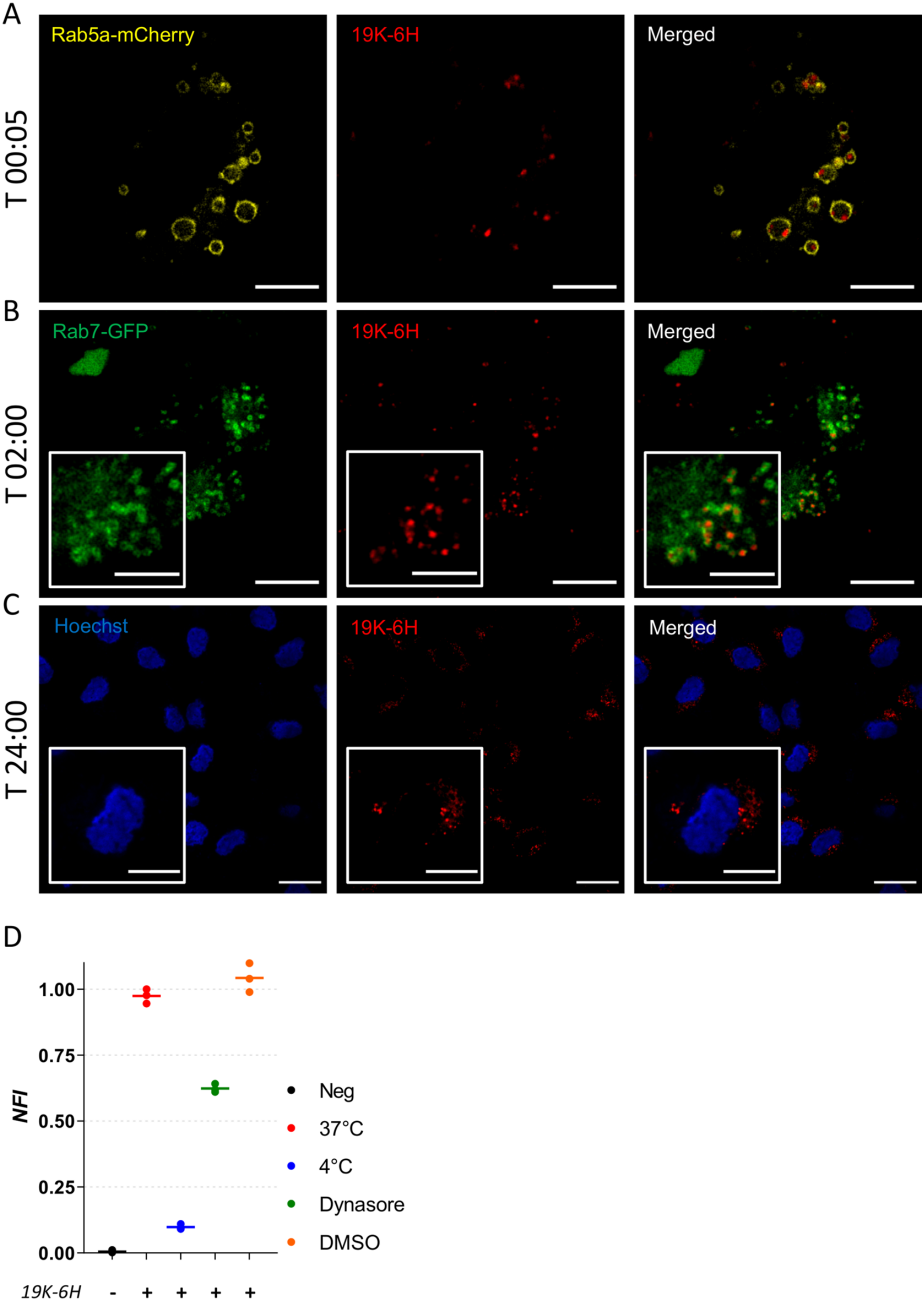
Confocal microscopy of living Hela cells incubated with 19K-6H probe and transfected with plasmid coding for endosomal markers fused with fluorescent proteins (Rab7-GFP, Rab5a-mCherry) or labelled with nuclear counterstain (Hoechst) (objective ×63). (**A**) HeLa cell transfected with Rab5a-mCherry plasmid, after 5 min incubation of 19K-6H probe (10 μM). Excitation 561 nm. Representative of 3 experiments. Scale bar: 10 μm. (**B**) HeLa cell transfected with Rab7-GFP plasmid, after 2 h incubation of 19K-6H probe (10 μM). Dual sequential excitation 488 nm and 561 nm. Representative of 3 experiments. Scale bar: 10 μm. ROI close up, scale bar: 5 μm. (**C**) HeLa cells labelled with Hoechst 33342, after 24 h incubation of 19K-6H probe (10 μM). Excitation 405 nm and 561 nm. Scale bar: 20 μm. ROI close-up, scale bar: 10 μm. Fluorescent images (visualized in blue, red, green and yellow: false colors) are presented separately on left and middle panel and merged on right panel. (**D**) HeLa cells are incubated 8 h with 19K-6H probe (10 μM) at 37 °C or 4 °C or in presence of 80 μM of Dynasore (37 °C) or DMSO (Dynasore Solvent—37 °C) in the culture medium. 19K-6H labelling is evaluated by FACS. NFI: normalized fluorescence intensity. n = 3. Bar: median values.

19K-6H-labeled pCD8 T cells displayed a heterogeneous subcellular distribution with vesicular spots of high fluorescence intensity, which suggested an internalization process via endocytosis. In order to investigate this possibility, we used a previously described model of transfected cells specifically expressing fluorescent proteins in the endosomal compartment^36,37^. HeLa cells were transfected with plasmids coding for early (Rab5a) or late (Rab7) endosomal markers fused with mCherry or GFP fluorescent proteins, respectively. 19K-6H probe was added 24 h after transfection and time-lapse acquisition was performed using spectral confocal microscopy. As shown in Fig. 4A, more than 95% of intracellular 19K-6H probe was found within Rab5a-mCherry positive early endosomes in the very first minutes after addition to the culture medium. After 2 h of incubation, the 19K-6H probe colocalized strongly with Rab7-GFP labelled late endosomes (Fig. 4B). Finally, even after a 24-h incubation, no 19K-6H fluorescence signal was detected in the nucleus of the HeLa cells (Fig. 4C). In a control experiment, endocytosis was then blocked either by incubating the cells at 4 °C or by adding Dynasore that inhibits dynamin, a key protein for several pathways of endocytosis, including clathrin-mediated endocytosis and other dynamin-dependent mechanisms^38^. 19K-6H internalization was reduced by 90% and 40% respectively, confirming that the probe is mainly internalized by endocytosis (Fig. 4D). The same experiment performed on pCD8 T cells gave similar results (Figure S7).

### Adoptive transfer of 19K-6H labelled pCD8 T cells in a mouse model of immune-mediated hepatitis

**Figure 5:**
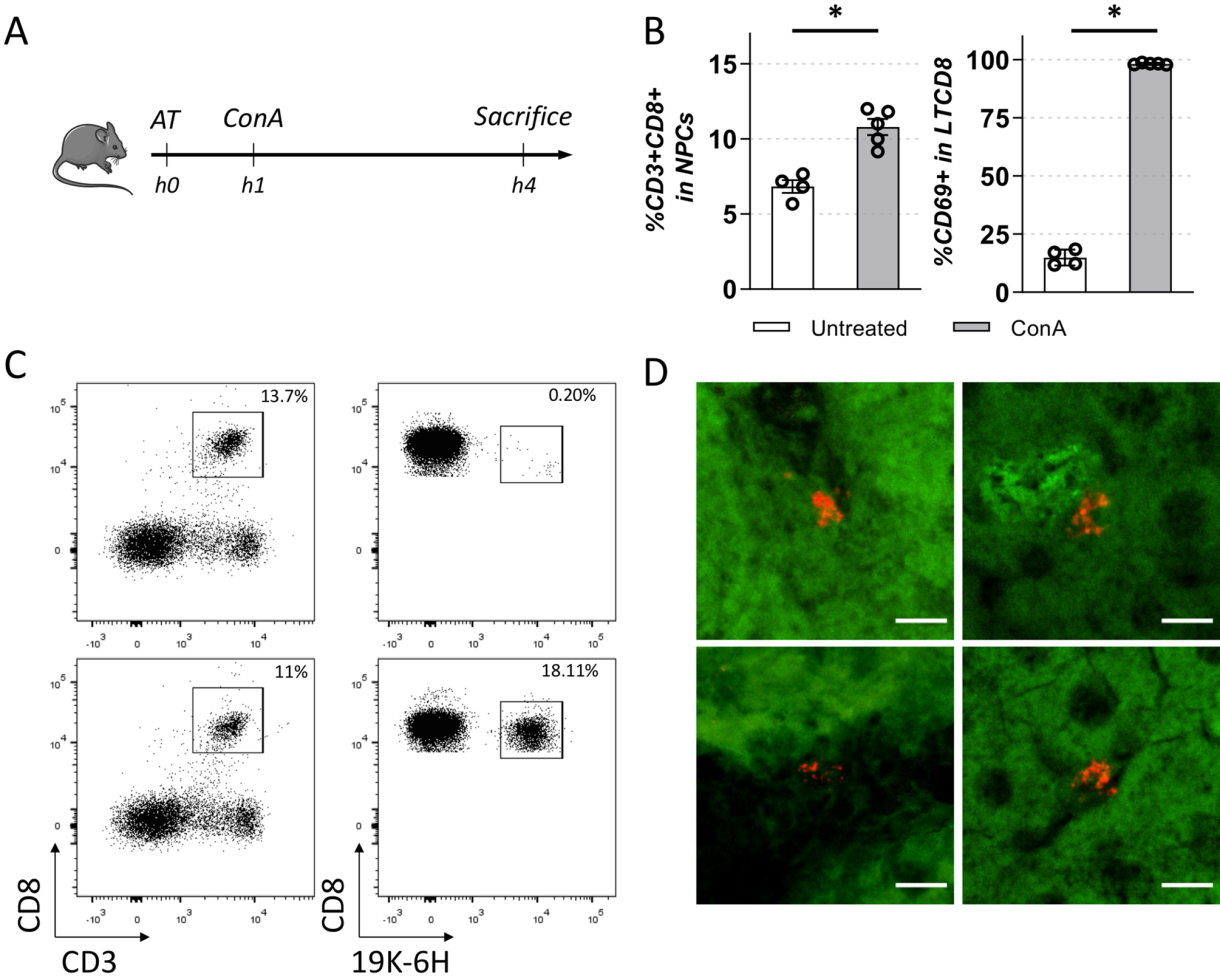
Adoptive transfer of pCD8 T cells (harvested the day before on C57Bl/6 mice, cultured O/N with activation signals) labelled or not with 19K-6H. (**A**) Mice are injected with 15 mg/kg Concanavalin A *(ConA)* or PBS 1 h after adoptive transfer *(AT)*, then sacrificed 3 h later. Liver are collected for (**B**) FACS analysis (CD3, CD8, CD69) of the recruitment of CD8 T cells (left panel) and their activation state (right panel) after ConA injection (significance is calculated by Mann–Whitney test. *p < 0.05), (**C**) FACS analysis (CD3, CD8, 19K-6H) of ConA treated mice, top panels: ctrl pCD8 T cells injected, bottom panels: 19K-6H-labelled pCD8 T cells injected and (**D**) confocal spectral microscopy analysis (objective ×20, scale bar 10 μm) on liver cryosections (thickness 15 μm) of 19K-6H-labeled pCD8 T cells/ConA injected mice. Excitation wavelengths were both 488 nm and 561 nm with 500–690 nm emission wavelength range (spectral imaging with 8.9 nm spectral band). Fluorescent images of 4 positives events are presented (false colors, green: liver background, red : 19K-6H positive events).

In order to demonstrate the interest of 19K-6H labelling for in vivo tracking of primary immune cells following their adoptive transfer, we used a classical mouse model of T cell-induced liver injury, in response to Concanavalin A (ConA) injection^39^. pCD8 T cells were prepared from donor mice, and incubated overnight with or without the 19K-6H probe (20 μM). 0.5 to 1.5 × 10^7^ pCD8 T cells (control or 19K-6H labelled) were injected intravenously to recipient mice, followed 1 h later by a single injection of ConA, or PBS (Fig. 5A). Mice were sacrificed 3 h later, and liver non-parenchymal cells (NPC) which include infiltrating leukocytes, were prepared and analysed by flow cytometry. Fragments of liver were also frozen and PFA-fixed for further confocal and TPEF-microscopy imaging, respectively. As expected, the ConA injection induced an enhanced recruitment of CD8 T cells to the liver, which are all in a CD69^+^ activated state, as shown by flow cytometry analysis (Fig. 5B). In mice that received ConA after adoptive transfer of 19K-6H-labelled pCD8 T cells, 18% of total liver CD8 T cells were 19K-6H positive (Fig. 5C). Confocal spectral microscopy analysis was performed on 15 μm frozen liver sections; far-red emitting cells were detected only in the liver of mice that had received 19K-6H labelled pCD8 T cells, however in very limited numbers (Fig. 5D). In order to acquire a higher number of events, and thanks to the high 2P-absorption of the 19K-6H probe, we then performed TPEF-microscopy for deep imaging.

**Figure 6:**
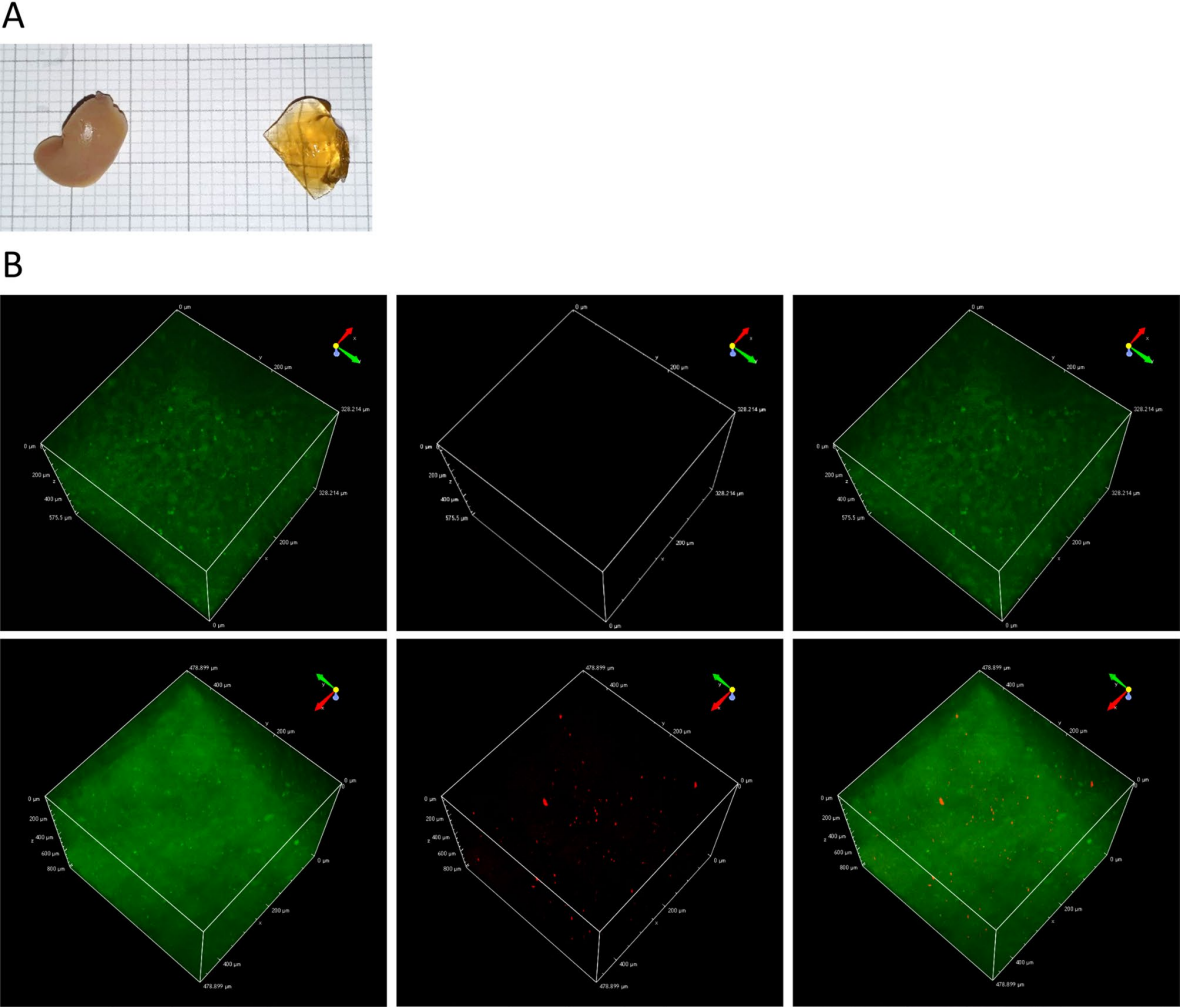
(**A**) Clearing of liver 800 μm sections thanks to CUBIC protocol. Left: before clearing; right : after clearing. (**B**) TPEF-microscopy analysis (objective ×25) on cleared liver 800 μm sections of 19K-6H-labelled (bottom panel—x: 478.9 μm, y: 478.9 μm, z: 800 μm) or control (top panel—x: 328.14 μm, y: 328.14 μm, z: 575.5 μm) pCD8 T cells injected mice. Dual wavelength excitation. From λ_ex_ 900 nm, autofluorescence was collected in green channel (500–550 nm). From λ_ex_ 1040 nm, 19K-6H fluorescence was collected in far-red channel (601–657 nm). Fluorescent images (visualized in green and red, false colors) are presented separately on left and middle panel and merged on right panel.

**Figure 7:**
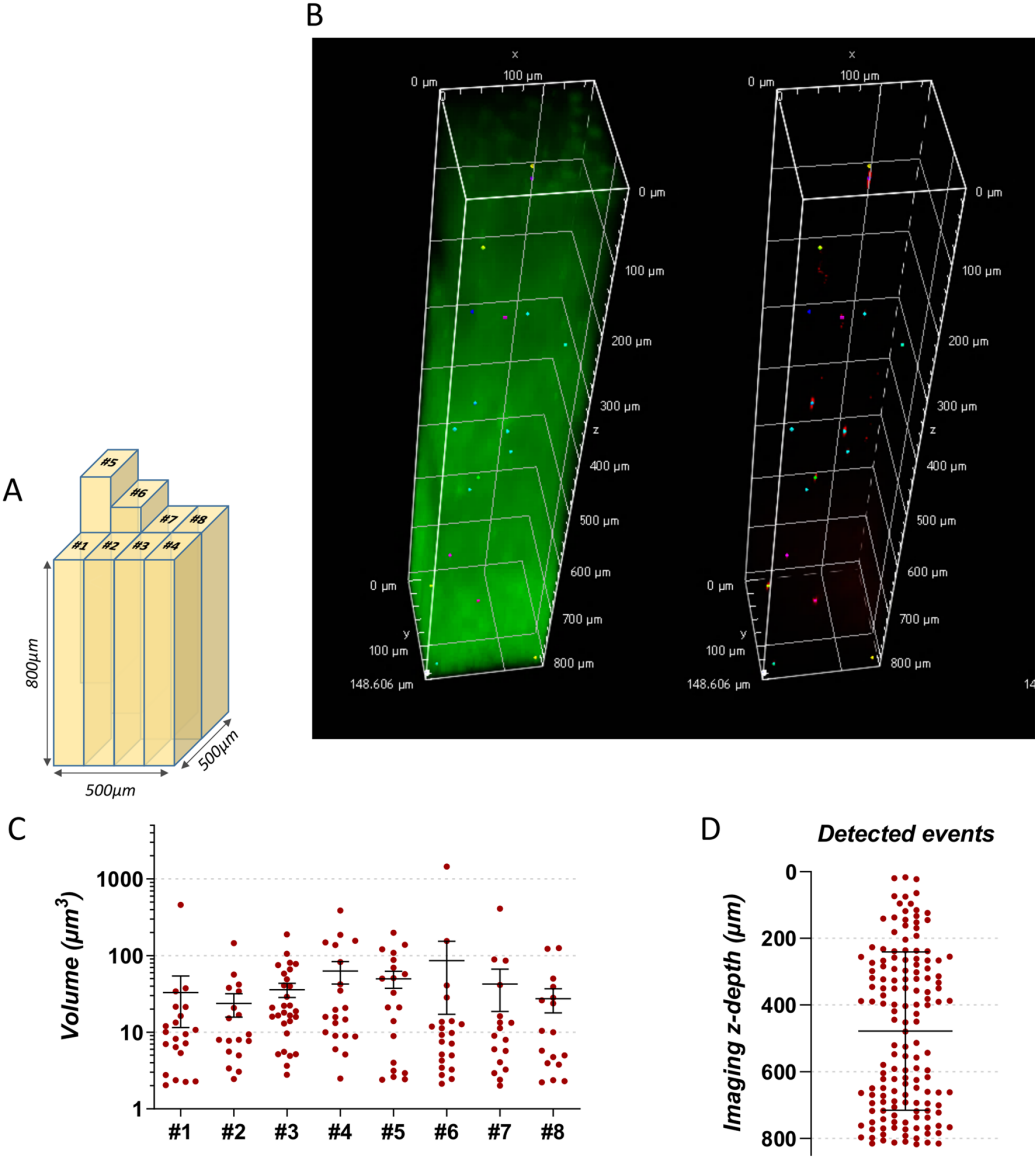
(**A**) 0.186 mm^3^ (~ 500 * 500 * 800 μm) of the liver of mice injected with labelled pCD8 T cells was investigated through 8 independent and non-overlapping analyses (#1 to #8) of 0.0232 mm^3^ with the (**B**) 3D measurement module included in NIS software. (**C**) Distribution of the far-red emitting events in the 8 independently analyzed liver volumes. Bars represent the mean and the standard-error calculated for each volume. (**D**) Distribution of the far-red emitting events in z-depth imaged in liver volume. Bars represent the mean and the standard-deviation.

TPEF-microscopy was carried out on 800 μm PFA-fixed liver sections, in order to get a more representative investigation of the pCD8 T cell infiltration within the liver. The sections were first cleared following CUBIC protocol^35,40^ to increase transparency (Fig. 6A), allowing a deeper (up to 4 mm) and less noisy imaging. Since the 19K-6H probe was efficiently excited at 1040 nm and enabled TPEF-imaging of labelled pCD8 T cells in vitro (Fig. 1C), the same wavelength was used to localize 19K-6H-labelled pCD8 T cells in 800 μm cleared liver sections and the 900 nm excitation wavelength was used to image the tissue autofluorescence. The individual images acquired from these 2 independent excitations (900 nm and 1040 nm) and collected in 2 independent channels (green and red channel respectively), and the overlay are presented on Fig. 6B. No liver autofluorescence was detected in red channel at 1040 nm excitation wavelength. For the liver of mice injected with 19K-6H-labelled pCD8 T cells, the reconstructed 3D image displayed a significant number of far-red emitting events (with variable volume and brightness), contrary to what was observed with the liver of mice injected with unlabelled pCD8 T cells (Fig. 6B). Quantification was carried out to confirm these observations and to characterize the detected events in terms of number, volume and distribution over the 900 μm depth of the sections. A total volume of 0.186 mm^3^ (500 * 500 * 800 μm) of the cleared liver of mice injected with labelled pCD8 T cells was probed by 8 independent and non-overlapping analyses (each of 0.0232 mm^3^—Fig. 7A), using the 3D measurement module included in NIS software (Fig. 7B).

A minimum event detection threshold of 2 μm^3^ was chosen according to the average volume of intracytoplasmic labelling (2 to 50 μm^3^ per cell) previously detected by confocal microscopy and to the mean volume of false-positive events detected in the control liver (0.20 μm^3^ ± 0.29). As a result, 148 far-red emitting events have been detected in the whole volume analysed of the positive liver, with a mean volume of 49 μm^3^ (± 134) with some objects reaching up to 1500μm^3^, suggesting concentration of several labelled cells. Distribution analyses demonstrated a homogenous repartition of the events in the 8 liver volumes independently analysed (mean events per volume = 9 ± 3, Fig. 7C) and also a homogenous repartition of the events in depth (Q1: 278.9 μm, median: 451.9 μm, Q3: 694.7 μm—Fig. 7D) thus eliminating a potential detection defect correlated to the imaging depth.

## Discussion

The development and optimization of primary cell tracking in animal models is of major importance in the field of cell therapy in order to better assess the engraftment properties, behaviour and localization of the injected cells. For this purpose, non-invasive strategies consisting in the ex vivo labelling of the cells with fluorescent probes before their injection has been developed in the last decade, as an alternative to genetic modifications, for instance^5,6^. The most important criteria for those probes are the biocompatibility (to preserve cell integrity and functionality, especially in the case of primary cells that are significantly more fragile than cell lines), the emitted fluorescence (intensity, stability, wavelengths, easy discrimination from tissue autofluorescence^8–10^) and the versatility for implementing multiscale technical approaches like microscopy and flow cytometry. In this regard, the development of fluorescent probes suitable for both confocal and TPEF-microscopy is of particular interest for deep imaging^11,12^. One of the challenging issues is to develop far-red emitting probes, highly discriminative from liver autofluorescence, exhibiting a large Stokes shift as well as high 2P-absorption and presenting biocompatibility properties at concentrations enabling a convenient imaging.

Water-soluble polymer probes bearing 2P-absorbing fluorophores emitting in the far-red range are excellent candidates. Previous results showed a low cytotoxicity on various cell lines^30^, with an efficient labelling of enveloped viruses^29^, living cells and zebrafish embryos enabling their visualization by TPEF-microscopy^30^. In this article, we investigated the benefits of the new 19K-6H far-red emitting polymer probe (Fig. 1A) for the tracking of primary immune CD8 T cells (pCD8 T cells) in a mouse model of liver infiltration through multiscale approaches including both confocal and TPEF-microscopy as well as flow cytometry for high-throughput cell analysis.

First, we demonstrated that pCD8 T cells were efficiently labelled with 5 to 20 μM of 19K-6H after 15-h incubation, resulting in an intracytoplasmic staining with vesicular spots of high intensity. The labelling was easily detected by confocal microscopy with 488 and 561 nm laser excitation wavelengths, and emission peaks exhibiting a maximum at 650 and 660 nm, respectively, have been determined with the spectral module. This result confirms previous observations and the large Stokes shift associated with the far-red emitting fluorophore covalently bound on the 19K-6H probe^27^. This large Stokes shift (around 180 nm, see Figure S2) enables minimization of the self-quenching effect and leads to an enhanced signal-to-noise ratio^41^. This represents a major advantage compared to most of the commercially available far-red emitting fluorophores; for instance: borondipyrromethene (BODIPY)^42,43^, cyanine or rhodamine dyes^44,45^, that display Stokes shifts limited to ~ 20 nm.

In addition, 19K-6H labelled-pCD8 T cells were optimally visualized under a 2P-excitation at 1040 nm, which is a very convenient wavelength in the range of standard femto-second pulse laser of common TPEF-microscopes. In a previous article^30^, after labelling HeLa cell lines with a related polymer probe bearing the same fluorophore, we determined a maximum 2P-excitation wavelength at 920 nm. However, in that experiment, the 2P-excitation spectrum could only be reconstructed up to 1000 nm due to the laser range limitation. Therefore, we may deduce that 1040 nm is the main maximum 2P-absorption wavelength of this polymer probe *in cellulo* and that 920 nm is a secondary maximum. It is worth mentioning that a 2P-absorption spectrum *in water* of an oligomer probe based on the same fluorophore indicated a maximum 2P-absorption wavelength around 1050 nm and a secondary maximum around 950–980 nm^28^.

We then investigated the stability of the 19K-6H labelling, in terms of fluorescence intensity and emission spectra, on murine pCD8 T cells and showed that the labelling was still detectable (by both confocal microscopy and flow cytometry) after 3 days of proliferation for the concentrations 5 μM and 20 μM. The fluorescence decrease, observed proportionally to the cell proliferation, argues for a fair partitioning of the 19K-6H probe during cell division, which is of importance considering the in vivo applications and the potential proliferation of the cells in the host. Furthermore, emission spectra were identical over time (Fig. 2D), indicating that the probes globally remained in the same local environment without degradation.

The working concentrations to obtain an adequate labelling for our application (5 to 20 μM) are higher than those reported for HeLa and Jurkat cells with a previous generation of far-red emitting polymer probes (0.2 to 1 μM)^30^. This could reflect the difference of brightness of those probes measured in water: 13100 cm^−1^ mol^−1^ L for the previous one^30^ and 2800 cm^−1^ mol^−1^ L for the present one. It could also be due to the intrinsically limited internalization process of primary cells compared to metabolically over-active tumoral cell lines. Indeed, when labelled simultaneously in the same conditions (5 μM 19K-6H, 15-h incubation), Jurkat cells showed a 4-time brighter labelling than primary CD8 T cells *(data not shown)*. Similar results have been described on HTB-125 (human epithelial non-cancerous cell) and HTB-126 (human epithelial cancerous cell) labelled with a lipid-derived fluorescent probe internalized through endocytosis^46^.

Primary cells present a high biological heterogeneity compared to established cell lines, more representative of their in vivo, physiological origin. It is thus necessary to study the potential cytotoxicity of the probe on the primary cells prior to develop in vivo cell tracking for cell therapy applications. In line with the innocuity of similar far-red emitting polymer probes previously shown on Jurkat cells^30^, we demonstrated in the present study that the 19K-6H probe did not induce direct cytotoxicity on primary CD8 T cells after a 15-h incubation even at the highest concentration (20 μM) (Fig. 3A). Such biocompatibility was further confirmed by the absence of long-term cytotoxicity, as neither the proliferation nor the activation of pCD8 T cells in response to mitogenic factors were altered (Fig. 3A). Thus, whereas the biocompatibility of many fluorescent nanoparticles remains to be thoroughly investigated^47^, we demonstrated here that the 19K-6H polymer probe labelling did not alter the function of the primary T cells.

Given the observed heterogeneous subcellular distribution of the 19K-6H probe in highly fluorescent spots which suggested a preferential localization in intracellular vesicles, we investigated the endocytosis-mediated internalization pathways with a previously described model of engineered HeLa cells^36,37^. The 19K-6H was found successively in early endosomes (Rab5A-mCherry, very first minutes after incubation) then in late endosomes (Rab7-GFP, 2 h) in a timely manner. Moreover, internalization was inhibited at 4 °C, a temperature that does not allow for endocytosis^48^. The partial internalization inhibition observed with Dynasore, a dynamin inhibitor^38^, suggested that the 19K-6H probe could take several endocytic pathways including dynamin-dependent and -independent pathways^49,50^. Therefore, the distribution of the probe during cell division could be linked to the stochastic endosome inheritance occurring during mitosis in mammalian cell lines^51,52^. As a perspective, it could be interesting to further investigate the fate of the probe in the cell, along the lysosomal and recycling endosomes pathways^53^.

Finally, we investigated the potential of the 19K-6H probe for the tracking of primary immune CD8 T cells in a mouse model of liver leukocyte infiltration. The development of a versatile fluorescent probe that can enable the use of several techniques within a single experiment, to bring complementary information is of high interest. In addition, this is also worthy on an ethical point of view, as it limits the number of animals for in vivo studies. Among the most informative techniques, the flow cytometry analysis enables a detailed high-throughput phenotyping of immune cells collected from an organ (such as the liver). Deep imaging with TPEF-microscopy brings the 3D localisation information which is lost in the tissue dissociation step necessary for flow cytometry analysis. We demonstrated here that the 19K-6H probe is very versatile as it can be used in the same experiment for both imaging of extemporaneously-labelled immune cells (from confocal microscopy to TPEF-microscopy for deep imaging) and flow cytometry. We used a well-designed mouse model of T cell-induced liver injury in response to Concanavalin A injection^39^. In this model, the injected 19K-6H labelled pCD8 T cells were easily detectable within the total NPC by flow cytometry but were seldom imaged by confocal spectral microscopy on 15 μm frozen liver sections. The 19K-6H-labelled pCD8 T cells could be imaged in vivo by TPEF-microscopy under 1040 nm excitation, the optimal 2P-absorption of the 19K-6H probe. With biological samples, TPEF-microscopy enables imaging on a maximal depth of 300 to 500 μm depending on the tissue^12,54^. To gain insight into the 3D localization of the labelled CD8 T cells within the liver, we associated TPEF-microscopy with a clearing protocol. The clearing protocol, applied as pre-treatment of the thick liver sections, increased imaging depth, and thus the probability of imaging relatively rare events. We used the CUBIC-protocol^16,35,40^ based on successive steps of delipidation, decolorization and refractive index matching, that allows deeper imaging (up to 4 mm) by lowering light scattering by the tissues. Moreover, this aqueous-based clearing protocol is supposed to be less toxic than organic ones and to improve fluorescence preservation^16^. TPEF-imaging could then be performed on 800 μm thick liver sections. 19K-6H-labelled pCD8 T cells appeared with a high signal-to-noise ratio in the far-red channel. This strong signal was highly specific, easily discriminated from the hepatic autofluorescence observed in the green channel. Indeed, by using adequate acquisition parameters and a fixed minimum threshold volume of 2 μm^3^, 148 far-red emitting events have been detected in the analysed liver sample (0.186 mm^3^) of mice injected with labelled pCD8 T cells whereas none were detected in the control liver (injected with unlabelled pCD8 T cells). Those events displayed a mean volume of 49 μm^3^ which is in line with the average size of pCD8 T cells (3–5 μm, Figs. 1B,D, 2B, S3, S4). The density of fluorescent events was quantified to 796 events/mm^3^, and it can be extrapolated to approximately 1.3 × 10^6^ 19K-6H-labelled pCD8 T cells for a whole liver (1650 mm^3^^55^). This range is consistent with the number of 19K-6H-labelled cells injected to the mice (1.5 × 10^7^ cells). In addition, the global homogenous distribution of the cells (Fig. 7B,C) with some events reaching up to 1500 μm^3^ (considered as cell clusters) match to the mouse model of T cell-mediated acute liver hepatitis induced by the Concanavalin A^39^.

In conclusion, we demonstrated in the present article that the new 19K-6H polymer probe is a highly-biocompatible and versatile probe with a bright and stable fluorescence adapted for multiscale and complementary approaches such as flow cytometry, for high-throughput cell analysis, and fluorescence microscopy (including TPEF-microscopy) for subcellular information and localization of the cells in the analysed organ. Moreover, we showed that the 19K-6H is a robust tool, resistant to acetone- and PFA-fixation as well as CUBIC-clearing, thus making it even more versatile and multiplying its potential fields of applications. Finally, given the high 2P-absorption of the 19K-6H probe at 1040 nm, another interesting application to investigate will be the intra-vital in vivo two-photon scanning laser microscopy by using dual wavelength excitation (1040/900 nm) to provide dynamic insights of cell behaviours in a real-time manner^14,15^.

## Supplementary information

Supplementary Information.
