# Supplementary material for: Multiscale fluorescent tracking of immune cells in the liver with a highly biocompatible far-red emitting polymer probe
Source: Sci Rep. 2020 Oct 16;10:17546. doi: 10.1038/s41598-020-74621-9 (PMC7567820; doi:10.1038/s41598-020-74621-9)
Supplement: Supplementary file 1 — Supplementary Information. [file 41598_2020_74621_MOESM1_ESM.docx]

Multiscale fluorescent tracking of immune cells in the liver with a highly biocompatible far-red emitting polymer probe.

**Malo DANIEL^1^, Laurence DUBREIL^2^, Romain FLEURISSON^2^, Jean-Paul JUDOR^1^, Timothée BRESSON^3^, Sophie BROUARD^1^, Arnaud FAVIER^3^, Marie-Thérèse CHARREYRE^3^, Sophie CONCHON^1^**

^1^Université de Nantes, INSERM, Centre de Recherche en Transplantation et Immunologie, UMR1064, ITUN, F-44000 Nantes, France.

^2^PAnTher, INRAE, École nationale vétérinaire, agro-alimentaire et de l’alimentation Nantes-Atlantique (Oniris), Université Bretagne Loire (UBL), Nantes F-44307, France.

^3^Laboratoire Ingénierie des Polymères (IMP), CNRS UMR5223, Université Lyon1, Université de Lyon.

**Supplementary information**

**Supplementary figures**

**Figure S1**

**Figure S1.** Chemical structure of the 19K-6H polymer probe. Blue : N-acryloylmorpholine (NAM) units; red: far-red emitting fluorescent units; green: negatively-charged sodium acrylate units.

**Figure S2**

**Figure S2.** Normalized absorbance (full line) and fluorescence emission (dashed line, λ_Ex_ = 510nm) spectra of the polymer probe in water (ε = 43400 m^-1^.cm^-1^ at λ_Abs max_ = 504 nm, φ = 0.064).

**Figure S3**

**Figure S3.** Fluorescence confocal spectral microscopy images (objective 63X - λ_ex_ 561nm) of pCD8 T cells labelled with 19K-6H probe (20μm, 15-hour incubation). Grey levels (8bits) were color-coded with the 16 colors look-up-table (LUT) from FIJI software. Collection of fluorescence emissions from 566 to 700nm with 15 steps of 8.9nm. Scale bar : 10μm.

**Figure S4**

**Figure S4.** Fluorescence confocal microscopy images (objective 63X) of proliferating pCD8 T cells (λ_ex_ 561nm) from day 1 to day 4 after 15 hours incubation of the 19K-6H probe (20μm). Grey levels (8bits) were color-coded with the 16 colors look-up-table (LUT) from FIJI software. Scale bar : 10μm.

**Figure S5**

**Figure S5.** Evolution of 19K-6H labelling on proliferating pCD8 T cells after 15 hours incubation of the 19K-6H probe. The labelling is monitored by flow cytometry (em710/50nm) on living cells, n=2. NFI: normalized fluorescence intensity.

**Figure S6**

**Figure S6.** Evolution of pCD8 T cells activation after 15 hours incubation with increasing concentrations (0.5; 5; 20μm) of 19K-6H probe compared to negative control (w/o 19K-6H). FACS analysis of 3 activation markers: early marker CD69 **(A)**, late markers CD44 **(B)** and CD25 **(C)**. The positive gates are determined thanks to negative control. Representative of 3 experiments.

**Figure S7**

**Figure S7.** pCD8 T cells are incubated 8 hours with 19K-6H probe (10μm) at 37°C or 4°C or in presence of 80μm of Dynasore (37°C) or DMSO (Dynasore solvent – 37°C) in the culture medium. 19K-6H labelling is evaluated by FACS. NFI: normalized fluorescence intensity. n=3. Bar : median values.
